# The modern “3G” age of archaeal molecular biology

**DOI:** 10.3389/fmicb.2012.00430

**Published:** 2012-12-20

**Authors:** Frank T. Robb, Todd M. Lowe, Zvi Kelman

**Affiliations:** ^1^Institute of Marine and Environmental TechnologyBaltimore, MD, USA; ^2^University of Maryland School of MedicineBaltimore, MD, USA; ^3^Department of Biomolecular Engineering, University of CaliforniaSanta Cruz, CA, USA; ^4^National Institute of Standards and TechnologyGaithersburg, MD, USA; ^5^Institute for Bioscience and Biotechnology ResearchRockville, MD, USA

The publication of the genome of *Methanocaldococcus jannaschii* in 1996 (Bult et al., 1996), just the second-ever complete microbial sequencing feat, marked an exciting beginning for archaeal genomics. In spite of the auspicious start, progress in archaeal genomics has been slow relative to bacteria and viruses, in part due to sequencing funding priorities favoring microbes with clinical relevance. In the last 5 years, the precipitous drop in high throughput sequencing costs and increasingly automated genome annotation has caused the inventory of archaeal genomes to grow exponentially. There is now genome representation in all major phyla of the Archaea, with a number of popular genera (including Halobacterium, Methanocaldococcus, Methanosarcina, Pyrobaculum, Pyrococcus, Sulfolobus, and Thermococcus) represented by multiple species. With these sequence data, comparative genomic studies have played an important role in integrating a broader evolutionary perspective into archaeal research. Experimental genetics now commonly utilize multiple archaeal species in a modern systems approach, coinciding with an increasing upswing in international collaboration. Multilaboratory consortia, such as the SulfoSys community (Albers et al., 2009), have started to break into new systems biology initiatives. At the same time, streamlined genetic approaches in the genera Halobacterium/Haloferax, Methanosarcina, Sulfolobus, and Thermococcus, are leveraging the growing wealth of genomic information in the Archaea. The result has been genome-driven studies of metabolism, DNA replication and repair, transcription and translation, and post-translational processing.

This collection of 11 papers attempts to put this quiet maturation in archaeal molecular biology in context with a mix of overview articles (Atomi et al., 2012; Hileman and Santangelo, 2012; Kohler and Metcalf, 2012; Wagner et al., 2012) and original data (Bernick et al., 2012a,b; Iverson and Stedman, 2012; Mao and Grogan, 2012; Schut et al., 2012; Stroud et al., 2012; Szabo and Pohlschroder, 2012) highlighting the expansion of important areas enabled by the 3G's: genomics, genetics, and global collaboration. Research papers highlight the roles of small RNAs, CRISPR action, mechanisms of membrane secretion, hydrogen production, and DNA replication, while excellent technical overviews cover recently developed genetic methods within four major research communities utilizing Haloferax, Sulfolobus, Pyrococcus, or Thermococcus.
